# Pre-conditioning with synthetic CpG-oligonucleotides attenuates myocardial ischemia/reperfusion injury via IL-10 up-regulation

**DOI:** 10.1007/s00395-013-0376-7

**Published:** 2013-08-09

**Authors:** P. Markowski, O. Boehm, L. Goelz, A. L. Haesner, H. Ehrentraut, K. Bauerfeld, N. Tran, K. Zacharowski, C. Weisheit, P. Langhoff, M. Schwederski, T. Hilbert, S. Klaschik, A. Hoeft, G. Baumgarten, R. Meyer, P. Knuefermann

**Affiliations:** 1 Department of Anaesthesiology and Intensive Care Medicine, University Hospital Bonn, Sigmund-Freud-Straße 25, 53127 Bonn, Germany; 2 Institute of Physiology II, University of Bonn, Nussallee 11, 53115 Bonn, Germany; 3 Department of Anesthesiology, Intensive Care Medicine and Pain Therapy, University Hospital Frankfurt, Theodor-Stein-Kai 7, 60590 Frankfurt am Main, Germany

**Keywords:** Preconditioning, CpG-ODN, Ischemia/reperfusion, Heart, TLR9, IL-10

## Abstract

The aim of the study was to investigate whether pre-conditioning with CpG-oligodeoxynucleotides (CpG-ODN) may change cardiac ischemia/reperfusion (I/R)-dependent inflammation and modulates infarct size and cardiac performance. WT and TLR9-deficient mice were pre-treated with 1668-, 1612- and H154-thioate or D-Gal as control. Priming with 1668-thioate significantly induced inflammatory mediators in the serum and a concomitant increase of immune cells in the blood and spleen of WT mice. Furthermore, it induced myocardial pattern recognition receptors and pro-inflammatory cytokines peaking 2 h after priming and a continuous increase of IL-10. 16 h after pre-conditioning, myocardial ischemia was induced for 1 h. Infarct size determined after 24 h of I/R was reduced by 75 % due to pre-conditioning with 1668-thioate but not in the other groups. During reperfusion, cytokine expression in 1668-thioate primed mice increased further with IL-10 exceeding the other mediators by far. These changes were observed neither in animals pre-treated with 1612- or H154-thioate nor in TLR9-deficient mice. The 1668-thioate-dependent increase of IL-10 was further supported by results of a micro-array analysis 3 h after begin of reperfusion. Block of IL-10 signaling increased I/R size and prevented influence of priming. In the group pre-treated with 1668-thioate, cardiac function was preserved 24 h, 14 days and 28 days after I/R, whereas animals without pre-conditioning exhibited impaired heart function 24 h and 14 days after I/R. The excessive 1668-thioate-dependent IL-10 up-regulation during pre-conditioning and after I/R seems to be the key factor for reducing infarct size and improving cardiac function. This is in agreement with the finding that IL-10 block prevents cardioprotection by pre-conditioning.

**Electronic supplementary material:**

The online version of this article (doi:10.1007/s00395-013-0376-7) contains supplementary material, which is available to authorized users.

## Introduction

Myocardial infarction (MI) is the result of an acute occlusion of a coronary artery due to plaque rupture and subsequent thrombosis [27]. The prognosis of patients suffering from MI is mainly determined by the infarct size (IS) [14], which in turn depends on the inflammatory response of the myocardium. It has been reported that preceding angina pectoris (AP) reduces the inflammatory response and thus attenuates subsequent ischemia/reperfusion (I/R) injury. This phenomenon termed ischemic pre-conditioning (IPC) has been confirmed in numerous animal models (for review see [12, 19]). Pre-conditioning (PC) can also be achieved by antecedent application of physical, e.g. hyper- or hypothermia or chemical stimuli, e.g. anesthetics and virulence factors [7, 21, 35]. PC of the heart has been reported to reduce IS to improve left ventricular function and to protect against lethal arrhythmia [17]. The actual knowledge of cardioprotection has been summarized in [3, 18].

The inflammatory response induced by I/R is mainly mediated by the innate immune system [11]. As part of innate immunity, pattern recognition receptors (PRRs), in particular Toll-like receptors (TLRs), have been linked to myocardial I/R [11]. TLRs are primarily expressed in immune cells, such as macrophages and dendritic cells. These immune cells, but also other cell types such as myocardial cells, are responsible for TLR signaling in the heart [25]. Especially, TLR2- and TLR4 deficiency as well as pre-conditioning with their respective ligands Pam3CSK4 and lipopolysacharide (LPS) has been shown to reduce myocardial infarct size [29, 39]. TLR stimulation as PC initially induces a transient up-regulation of inflammatory mediators, which in turn may modulate the subsequent inflammatory response and desensitize the heart to I/R [11]. On the other hand, I/R by itself is supposed to release endogenous ligands that bind as damage associated molecular patterns (DAMPs) to TLR2 and TLR4 [8, 47]. Finally, TLR deficiency as well as PC via TLR2, -4 and -9 stimulation converges in a diminished inflammatory response leading to reduced IS [28, 29, 45]. With respect to potential clinical application, TLR2 and TLR4 ligands, e.g. LPS, seem rather unsuitable for the use in humans due to serious side effects. However, synthetic TLR9 ligands such as CpG-oligodeoxynucleotides (CpG-ODNs) also induce a strong innate immune response with less harmful side effects for the human organism [24]. Thus, CpG-ODNs are currently applied in clinical trials as immunostimulatory agents as well as vaccine adjuvants [4, 32]. The immunostimulatory potential of CpG-ODNs depends on the CG-motif as well as on the degree of methylation, e.g. 1668-thioate is a stimulatory ODN. In addition, suppressive ODNs such as H154-thioate have the potential to specifically inhibit the immune activation and do not induce an inflammatory response [10, 41]. It has already been shown that priming with CpG-ODNs is able to protect the heart against the harmful consequences of hypertension as well as ischemia [7, 28, 44]. Pathway analysis of the TLR9-dependent innate immune response revealed an up-regulation of interferon-γ and cellular growth signaling during the priming phase as well as an up-regulation of pro- and anti-inflammatory cytokines [44]. Interestingly, it has recently been shown that IL-10 is able to attenuate I/R injury in the murine heart [5]. This can be taken as a sign that the activation of anti-inflammatory pathways is able to interfere with I/R injury.

Therefore, we tested the hypothesis whether the systemic inflammation caused by CpG-ODN pre-conditioning may change the I/R-dependent cardiac inflammation and whether this in turn is correlated to variations of infarct size and cardiac performance. As changes in inflammation due to I/R have to be separated from surgery-induced trauma, all experiments were performed in a closed-chest model as best approach to the clinical situation.

## Materials and methods

All mice were handled in accordance with the Guide for Use and Care of Laboratory Animals (NIH publication No. 85-23, revised 1996). Protocols were approved by the local authority (LANUV, Recklinghausen, Germany).

### Surgical techniques, experimental groups and protocols

**Fig. 1:**
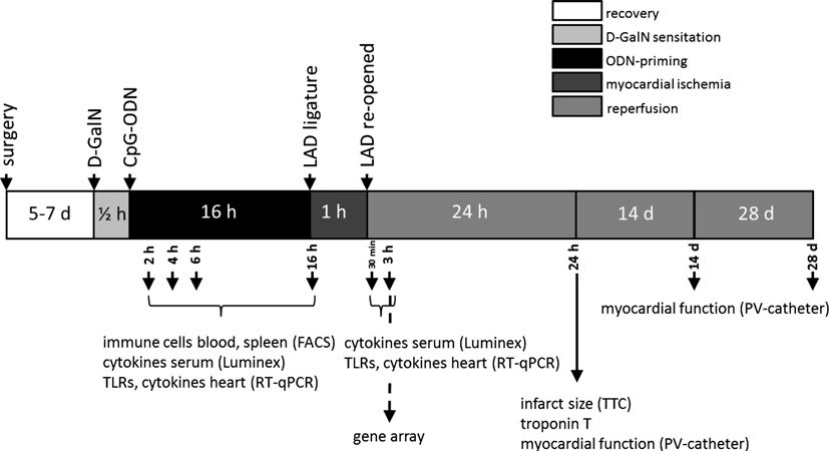
Time course of experimental interventions and assessments. Non-linear scale for better identification of various interventions

10 to 12-week-old male C57BL/6 wild-type (WT; Charles River Laboratories) and TLR9-deficient (TLR9-D) mice (kindly provided by Professor Shizuo Akira, Osaka University, Osaka, Japan; back-crossed to a C57BL/6 background) were included. To prevent an inflammatory reaction due to surgical trauma, a chronic closed-chest model of myocardial I/R with left parasternal incision was applied. Surgical procedures and assessment of IS are described and depicted in Kim et al. [22] and in the supplement. After 5–7 days of recovery, all mice were treated with an intraperitoneal (i.p.) dose of d-GalactosamineN (d-GalN, 0.2 mg/g, Roth, Karlsruhe, Germany) to slow down the hepatic degradation of CpG-ODNs (Fig. 1) [38]. In control experiments, d-GalN alone did not induce an inflammatory response (data not shown). Animals were randomized to intraperitoneally receive a stimulatory CpG-ODN (1668-thioate; 5′-TCC-ATG-A*CG*-TTC-CTG-ATG-CT; TibMolBiol, Berlin Germany; 0.25 nmol/g), a non-CG containing ODN (1612-thioate; 5′-GCT-AGA-TGT-TAG-CGT5; TibMolBiol; 0.25 nmol/g), a suppressive ODN (H154-thioate; 5′-CCT-CAA-GCT-TGA-GGG-G-3; TibMolBiol, Berlin Germany; 0.25 nmol/g) or d-GalN as control. In pre-experiments, the dosage of CpG-ODN 1668-thioate was tested (0.05, 0.25, 0.5 and 1.0 nmol/g). Mortality during I/R experiments was around 15 %. This was not influenced by priming with 0.05 and 0.25 nmol/g but the higher doses increased mortality. 16 h after ODN-priming myocardial infarction was induced for 1 h, followed by 24 h, 14 or 28 days of reperfusion (for time course see Fig. 1). 16 h were chosen because we knew from earlier experiments that pre-conditioning with LPS leads to a reduction of infarct size after this time span [39] and that priming with CpG-ODN 1668-thioate applied under the same conditions (interval and concentration) as used here was able to attenuate cardiac hypertrophy induced by transverse aortic constriction [44].

Two further groups (with and without ODN-priming) of mice were treated i.p. with an anti-mouse monoclonal IL-10R1 antibody (5 μg diluted in 200 μl PBS; Biolegend, San Diego, CA, USA) directly after ODN-priming and 30 min prior to ischemia to block the influence of this anti-inflammatory cytokine during preconditioning and I/R [5].

### Troponin T-measurement

350 μl blood was collected from the right carotid artery. The blood samples were analyzed with a Cardiac Reader^®^ (Roche, Grenznach, Germany).

### Multiplex cytokine assay

Blood was collected 2, 4, 6 or 16 h after priming and 30 min or 3 h after reperfusion. Blood was centrifuged at 3,400*g* for 10 min and the supernatant (plasma) was stored at −20 °C. After thawing, samples were analyzed immediately at 37 °C. Levels of TNF-α, IL-1β, IL-6, IL-10, IL-12, and IFNγ (Mouse Cytokine multi-Plex for Luminex™ laser, BioSource Europe) were determined using the microsphere array technique (Luminex 100 system, Luminex Corporation, Austin, TX, USA) as previously described [25].

### Flow cytometry analysis of immune cells in the spleen and blood

Single cell suspensions of the spleens were generated by digestion with 1 mg/ml collagenase IV (Sigma, Taufkirchen, Germany) and 50 U/ml DNaseI (Sigma) in PBS with 3 % FCS for 30 min at 37 °C under mild stirring. Blood was collected by cardiac puncture in the presence of 0.5 M EDTA. One volume of blood was mixed with 20 volumes of Red Cell Lysis Buffer (145 mM NH_4_Cl, 100 μM EDTA, 12 mM NaHCO_3_). Spleen and blood cells were incubated with clone 2.4G2 culture supernatant to block Fc receptors. The following labeled antibodies from Pharmingen (BD Biosciences, San Diego, CA, USA) or eBiosciences (San Diego, CA, USA) were used at 1:200 dilution for staining of 1 × 10^6^ cells: Gr1 PerCP-Cy5.5 (RB6-8C5), CD11c FITC (HL-3), CD11b APC (M1/70) and F4/80 PE (BM8). Dead cells were excluded with Hoechst 33342 stain [42, 43]. Sorting was performed on a FACSDiva cell sorter (BD Biosciences).

### RNA-extraction and Taqman^®^ RT-qPCR

The mRNA expression levels of TLR2 and -9 and as well as TNF-α, IL-1β, IL-6, and IL-10 were determined using Taqman^®^ Realtime quantitative PCR (RT-qPCR; Applied Biosystems, Darmstadt, Germany). Total RNA was isolated with a standard protocol (Qiagen, Hilden, Germany). First-strand cDNA was synthesized using the High Capacity cDNA transcription kit (Applied Biosystems, Darmstadt, Germany) with random hexameric primers as described in the manufacturer’s protocol. RT-qPCR was performed and analyzed with 1/10 diluted cDNA according to the manufacturer’s instructions on an ABI Prism 7900 Sequence Detection System and SDS 2.2 Software (Applied Biosystems). Target gene expression was normalized to an internal control (glyceraldehyde-3-phosphate dehydrogenase, GAPDH). Relative RT-qPCR was performed using TaqMan Gene expression Master Mix (part 4369016; Applied Biosystems) with the following primers: GAPDH (Mm99999915_g1), TLR2 (Mm01213945_g1), TLR9 (Mm00446193_m1), IL-1β (Mm99999061_g1), IL-6 (Mm01210732_g1), IL-10 (Mm00439616_m1) and TNF-α (Mm00443258_m1). All murine primers were measured using FAM TAMRA chemistry and the relative standard curve method. At the end of the RT-qPCR cycle, dissociation curve analysis was performed to ascertain the amplification of a single PCR product.

### Gene-expression analysis

Microarray analysis was used to monitor gene expression in the heart 30 min and 3 h after begin of reperfusion.

#### Microarray hybridization

Cy3 labeled reference and Cy5 labeled sample cDNAs (10 μl each) were combined, denaturated by heating for 2 min at 98 °C and mixed with 36 μl of hybridization solution at 42 °C (Ambion, Austin, TX, USA). Murine microarrays (NimbleGen, Roche, Basel, Switzerland) were overlaid with this solution and hybridized for 18 h at 42 °C using an actively mixing MAUI hybridization system (BioMicro Systems, Salt Lake City, UT, USA). Post hybridization the arrays were washed in 1× SSC/0.05 % SDS and 0.1× SSC, centrifuged to remove remaining liquid with unbound cDNA, and dried. Intensity values were generated using an array scanner (NimbleGen). Data were up-loaded to the mAdb database (Microarray Database, a collaboration of CIT/BIMAS and NCI/CCR at the NIH; http://nciarray.nci.nih.gov/) and formatted via the export function for use with BRB ArrayTools (Biometric Research Branch, NCI, Frederick, MY, USA).

#### Analysis of gene expression

Data from three independent experiments/time points and three untreated controls were used for statistical analyses. Expression analyses were performed using BRB ArrayTools. Data were background corrected, flagged values were removed, spots in which both signals were <100 were filtered out, ratios were log base 2 transformed and lowess intensity-dependent normalization was used to adjust for differences in labeling intensities of the Cy3 and Cy5 dyes [46]. Analysis was restricted to genes present on >50 % of the arrays after filtering. The gene expression profile of all treatment groups was compared to that of the control groups. A *p* value cutoff of 0.0001 was used to identify genes, whose expressions were significantly up-regulated after CpG-ODN stimulation when compared to controls. Data were evaluated using Ingenuity Pathway Analysis (IPA, Ingenuity Systems Inc., Redwood City, CA, USA). IPA maps each gene within a global molecular network, pathways and functional groups developed from information contained in the Ingenuity Pathways Knowledge Base (see: https://analysis.ingenuity.com/pa/info/help/Ingenuity_Network_Algorithm_Whitepaper_FINAL(2) [6]).

### Cardiac pressure–volume measurements

Measurements of cardiac function were performed with a 1.4 french pressure conductance catheter (SPR-839, Millar Instruments, Houston, TX, USA) 24 h, 14 and 28 days after reperfusion. Recordings were performed under reduced isoflurane anesthesia (1 vol%) and controlled body temperature. For detailed description, see [44].

### Statistics

Data are presented as mean ± SEM. Statistical analysis was performed by Student’s unpaired *t* test used to compare means between groups. Groups larger than two were analyzed with one-way ANOVA with Newman–Keuls post hoc test using GraphPad PRISM5 (La Jolla, CA, USA). *p* < 0.05 was considered statistically significant. For microarray analysis, genes that were differentially expressed in the treatment groups were identified using a random-variance *t* test with a significance level of *p* < 0.0001.

## Results

### Pre-conditioning with CpG-ODNs

#### Inflammatory mediators in the serum

**Fig. 2:**
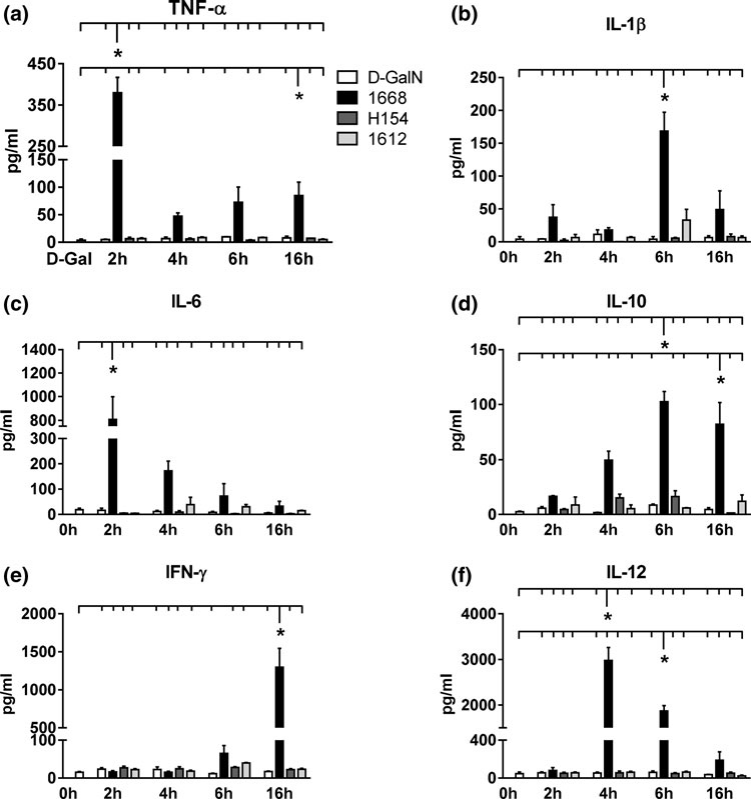
Cytokine (**a**–**e** TNF-α, IL-1β, IL-6, IL-10, IL-12) and IFNγ (**f**) protein in blood serum measured at 2, 4, 6 and 16 h after priming with 1668-thioate, H154-thioate and 1612-thioate. All investigated mediators were up-regulated during the priming phase by 1668-thioate stimulation, whereas the other CpG-ODNs did not affect protein levels in the serum (*n* = 5/group, *p < 0.05 *significant group)

1668-thioate significantly increased protein content in the serum of all determined inflammatory mediators during the priming phase, whereas stimulation with H154- and 1612-thioate did not change protein content of any of the investigated mediators. 1668-thioate up-regulated protein content of TNF-α (Fig. 2a), IL-1β (Fig. 2b), IL-6 (Fig. 2c), IL-12 (Fig. 2f) transiently with TNF-α and IL-6 reaching a maximum level after 2 h, IL-12 after 4 h and IL-1β after 6 h. IL-10 rose continuously reaching a plateau at 6 h (Fig. 2d). IFNγ production at least in part depends on IL-12, which rises 6 h after priming reaching the level of significance at 16 h (Fig. 2e). Pro-inflammatory mediators (IL-1β, IL-6, IL-12) had nearly returned to baseline level just before ischemia/reperfusion with the exception of TNF-α and IFNγ. In contrast, the anti-inflammatory mediator IL-10 had reached a high level at the start of cardiac injury. To prove TLR9-dependency of 1668-thioate stimulation, inflammatory parameters were also recorded in the serum of TLR9-D mice. None of the investigated mediators could be induced in TLR9-D animals (Supplement Figure 1).

#### Inflammatory cells in serum and spleen

**Fig. 3:**
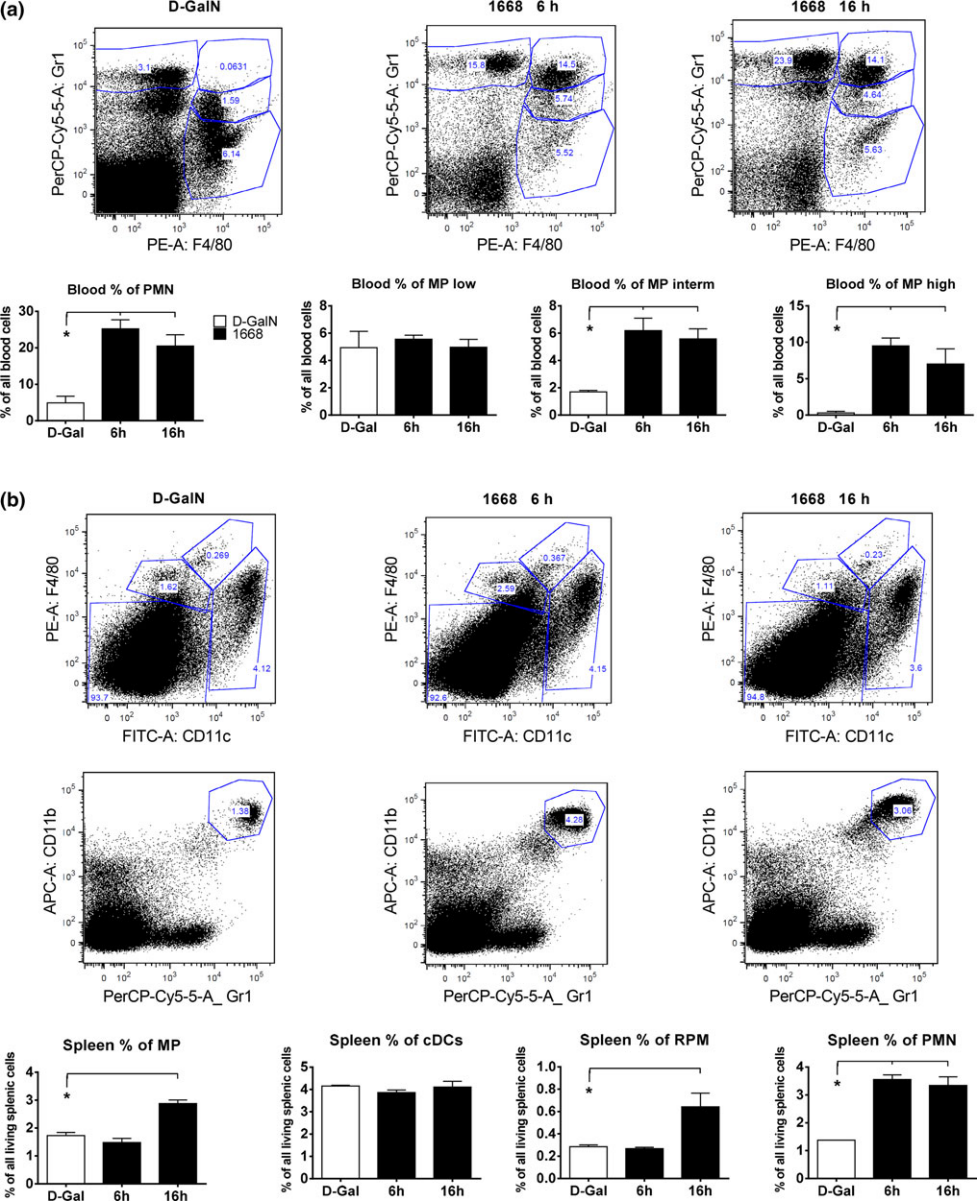
Flow cytometry of blood (**a**) and spleen (**b**) cells 6 and 16 h after priming with 1668-thioate and after d-GalN application. **a** Representative dot plots show the distribution of blood polymorph nuclear cells (PMN) as well as Gr1^low^, Gr1^intermediate^ and Gr1^high^ macrophages (MP) and *bar graphs* depict the quantitative evaluation of cell types 6 and 16 h after priming. **b** Representative dot plots and part of the gating tree used for detection of splenic MP, dendritic cells (cDC), red pulp macrophages (RPM) and PMN. Quantitative evaluation of MP and RPM is demonstrated in *bar graphs* 6 and 16 h after 1668-thioate stimulation (*n* = 4/group; *p < 0.05)

To further characterize the systemic inflammation caused by pre-conditioning, inflammatory cells were analyzed by flow cytometry of serum and spleen 6 and 16 h after stimulation (Fig. 3a, b). The number of polymorphous nucleic cells (PMN) in the serum significantly rose fivefold within the first 6 h and stayed high until 16 h (Fig. 3a). Macrophages (MPs) in the serum were divided into three groups Gr1^low^, Gr1^interm^ and Gr1^high^. Gr1^low^ macrophages were not influenced by priming, while the number of Gr1^interm^ and Gr1^high^ macrophages increased significantly by 3- and 30-fold, respectively, within the first 6 h remaining high until 16 h (Fig. 3a). In the spleen, the number of macrophages rose significantly by a factor of 1.5 between 6 and 16 h after stimulation (Fig. 3b). Splenic conventional dendritic cells (cDCs) proved to be insensitive to stimulation with 1668-thioate. Red pulp macrophages (RPM) rose twofold according to the same time pattern as MPs; however, their general number was low. Splenic PMNs became up-regulated within the first 6 h after stimulation and stayed elevated until 16 h (Fig. 3b). Taken together, PMNs as well as MPs in serum and spleen were significantly elevated at the onset of ischemia due to priming with 1668-thioate.

#### PRRs and cytokines in the myocardium

**Fig. 4:**
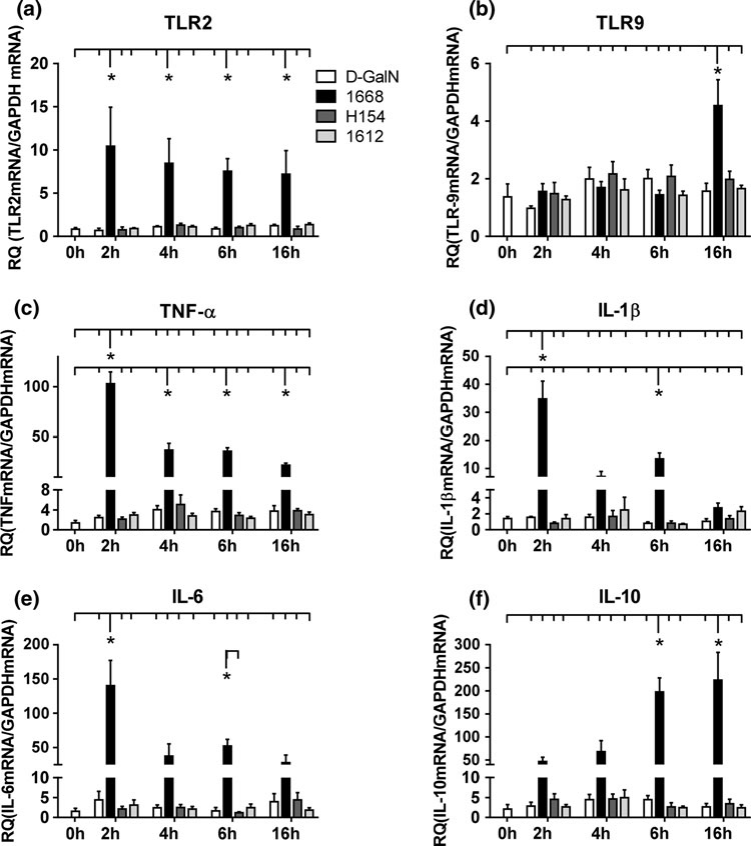
RT-qPCR of mRNA expression of PRRs (**a**, **b**) and cytokines (**c**–**f**) in the heart measured at 2, 4, 6 and 16 h after priming with 1668-thioate, H154-thioate and 1612-thioate. Stimulation with 1668-thioate differentially up-regulated both PRRs and all cytokines during the priming phase (*n* = 8/group, *p < 0.05; *significant group)

In line with the analysis of the blood serum, cytokine mRNA expression was analyzed in cardiac tissue; in addition, the expression of TLR2 and TLR9 was monitored (Fig. 4a–f). 1668-thioate injection resulted in a significant increase of all parameters during the priming phase with TLR2 (Fig. 4a), TNF-α (Fig. 4c), IL-1β (Fig. 4d) and IL-6 (Fig. 4e) peaking already after 2 h. TLR9 (Fig. 4b) expression reached the level of significance not until 16 h after 1668-thioate application. Similar to blood serum, IL-10 (Fig. 4f) continuously rose during the priming phase reaching a 200-fold increase after 16 h. Neither 1612-thioate nor H154-thioate was able to increase the mRNA expression of any inflammatory mediator. None of the genes of interest was influenced by CpG-ODN stimulation in TLR9-D mice at any time point (Supplement Fig. 2). Taken together, TLR2, TLR9, TNF-α and IL-10 were still elevated above control at the onset of cardiac ischemia.

### Ischemia–reperfusion-dependent changes

#### Infarct markers and inflammatory mediators in the serum

**Fig. 5:**
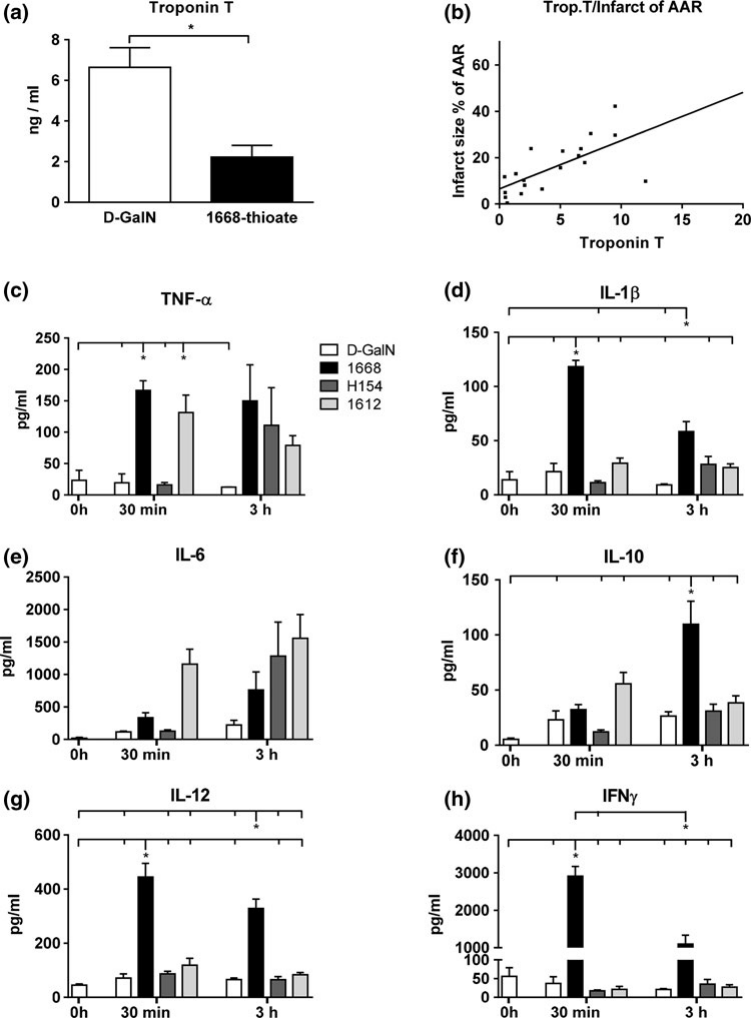
Recordings of different markers in the serum after ischemia/reperfusion. **a** Troponin T was measured 24 h after reperfusion. Priming with 1668-thioate significantly reduced serum troponin T. **b** Plot of serum troponin T (values taken from A) versus infarct size. (**a**, **b** slope: 2.081 ± 1.170E−008; correlation-coefficient: *r* = 0,67; *n* = 11/10, *p < 0.05). **c**–**h** Cytokine (**c**–**g** TNF-α, IL-1β, IL-6, IL-10, IL-12) and interferon-γ (**h**) protein in blood serum measured at 30 min and 3 h after reperfusion (*n* = 8/group, *p < 0.05; two * indicate that two groups differ significantly from the other marked groups)

In clinical practice, troponin T concentration in the serum is taken as a marker of myocardial injury. Therefore, troponin T concentration in murine serum was measured 24 h after reperfusion (Fig. 5a, b). The average troponin T concentration in 1668-thioate pre-conditioned mice was about three times smaller than in control animals pointing towards a decreased myocardial IS. To investigate whether troponin T may be employed as a marker of infarct size in small mammals such as mice, the troponin T concentration was plotted against infarct size (Fig. 5b). The correlation analysis revealed a linear slope of 2.081 ± 1.170E−008 with a correlation-coefficient of *r* = 0.67.

I/R injury caused a local inflammation in the myocardium, which may lead to a transient systemic inflammation. Therefore, inflammatory markers were monitored in the serum 30 min and 3 h after re-opening of the LAD (Fig. 5c–h). Interestingly, TNF-α (Fig. 5c), IL-1β (Fig. 5d), IL-6 (Fig. 5e), IL-10 (Fig. 5f), Il-12 (Fig. 5g), and IFNγ (Fig. 5h) were not significantly increased at both time points in the d-GalN group. However, all parameters except IL-6 were significantly elevated in the 1668-thioate primed mice. Surprisingly, TNF-α was also increased in the 1612-thioate pre-treated animals, whereas H154-thioate and I/R did not cause significant changes.

#### Infarct size, PRRs and cytokines in the left ventricle

**Fig. 6:**
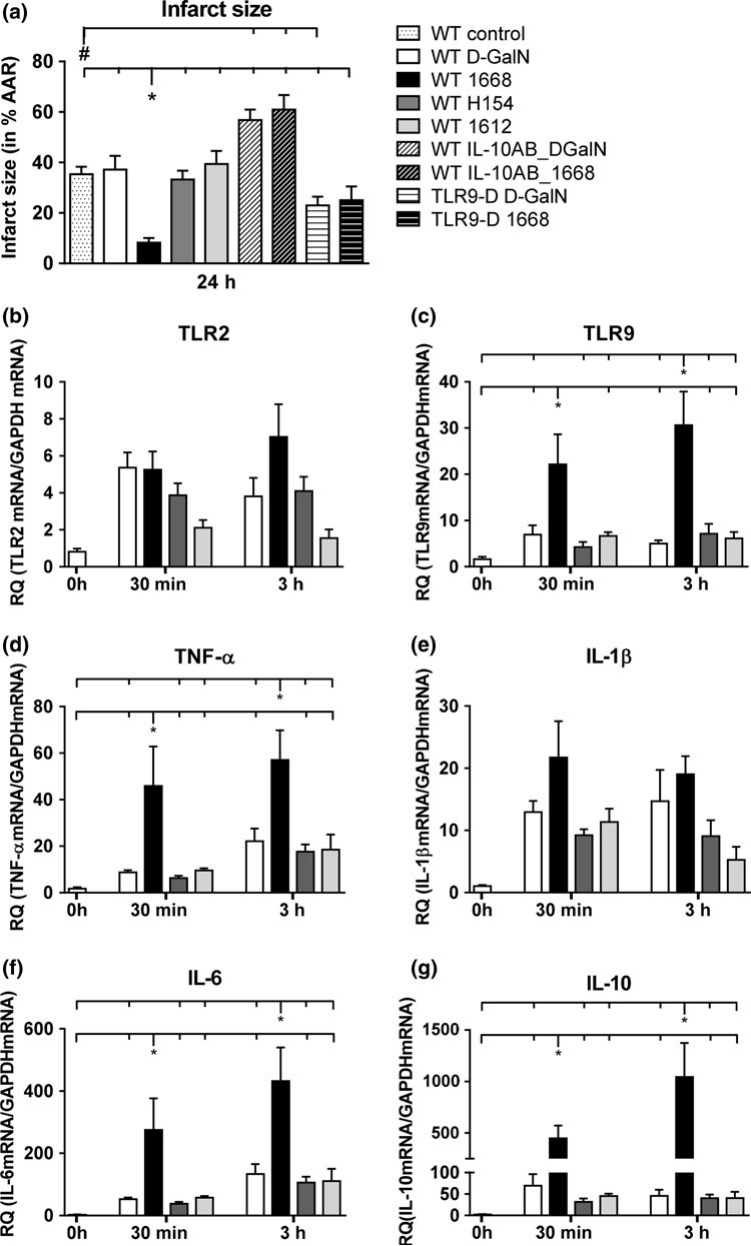
Recordings of infarct size (IS) and PRRs as well as cytokines from the heart after ischemia/reperfusion. **a** IS in percent of area at risk (AAR) evaluated by TTC staining after 24 h of reperfusion. Priming with 1668-thioate significantly reduced IS. Blocking with IL-10 antibody effectively blocked protection by 1668-thioate priming. IS in TLR9-D mice was significantly lower than in WT mice. Priming of WT animals with 1668-thioate reduced IS to a higher degree than TLR9-deficiency. Priming of TLR9-D mice with 1668-thioate was ineffective (*n* = 8/group, *p < 0.05 vs. WT 1668; ^#^ *p* < 0.05 vs. WT control). RT-qPCR of mRNA expression of PRRs (**b**, **c**) and cytokines (**d**–**g**) recorded at 30 min and 3 h of reperfusion. Priming with 1668-thioate resulted in a significant increase of TLR9, TNF-α, IL-6 and IL-10 mRNA expression at both time points (*n* = 8/group, *p < 0.05; *significant group)

Infarct size in d-GalN or H-154-thioate and 1612-thioate pre-treated animals was around 35–40 % of AAR 24 h after reperfusion (Fig. 6a), whereas infarct size of 1668-thioate pre-treated animals stayed below 10 % of AAR, i.e. infarct size of 1668-thioate primed mice was less than a third of that of control animals (Fig. 6a). This is in agreement with the troponin T concentration in the serum (Fig. 5a). To demonstrate the TLR9-dependency of reduced infarct size, TLR9-D animals had also been pre-treated with 1668-thioate. TLR9-deficiency itself resulted in a significantly blunted infarct size of about 23 % of AAR, which could not be further reduced by 1668-thioate pre-treatment in these mice (Fig. 6a).

Messenger RNA expression of markers of cardiac inflammation was monitored 30 min and 3 h after reperfusion (Fig. 6b–g). In accordance with findings in blood serum, I/R alone did not result in an increased expression of these markers in the myocardium. However, TLR9 (Fig. 6c), TNF-α (Fig. 6d), IL-6 (Fig. 6f) and IL-10 (Fig. 6g) were clearly and significantly up-regulated 30 min and 3 h after I/R in the 1668-thioate primed group. Interestingly, extremely high values were reached in case of IL-6 and IL-10. To control whether these high levels of IL-10 are involved in the beneficial influence of ODN-priming, we blocked the IL-10 receptor, IL-10R1, with a specific antibody in WT animals with and without priming. Blockade of the IL-10R1 led to a significant increase of infarct size. In the mice without priming, the infarct size amounted to 57 % of AAR after IL-10R1 blockade compared to 35 % with intact IL-10 signaling (Fig. 6a). In case of 1668-thioate priming, IL-10R1 inhibition resulted in an infarct size of 61 % of AAR compared to 8 % without antibody.

#### Micro-array analysis of the myocardium

For a more detailed analysis, micro-array investigations were performed on myocardial tissue 4 h after pre-conditioning as well as 30 min and 3 h after reperfusion. All treatment groups were normalized to untreated, healthy mice. During pre-conditioning phase, mice pre-treated with 1668-thioate were compared to sham mice whereas after reperfusion four groups (d-GalN alone, sham and 1668-, 1612-thioate pre-treatment) were investigated. 4 h after priming, the IL-10 pathway expression revealed a clear, albeit less prominent up-regulation than the IFN pathway (IL-10 pathway: *p* < 2.1E−3 vs. IFN signaling pathway: *p* < 1.2E−11). We also evaluated the expression of NF-κB pathway inhibitors TNFAIP3, NFKBIA, TRIM30 and TNIP1, most of which were up-regulated (TNFAIP3: 3.0; NFkBIA: 3.7; TRIM30: 5.04; TNIP1: 0.0).

During reperfusion, sham operation did not induce gene expression above a random distribution at the time of reperfusion. I/R itself (d-GalN) increased expression in a relatively small number of genes (30 min: 10 genes, 3 h: 76 genes, respectively), which is in accordance with the RT-qPCR results on inflammatory mediators. Pre-treatment with 1612-thioate only moderately increased gene expression in the heart after I/R (30 min: 50 genes; 3 h: 131 genes). On the other hand, 1668-thioate pre-treatment resulted in an up-regulation of 635 genes 30 min after I/R, increasing further towards 3 h with 697 up-regulated genes.

**Fig. 7:**
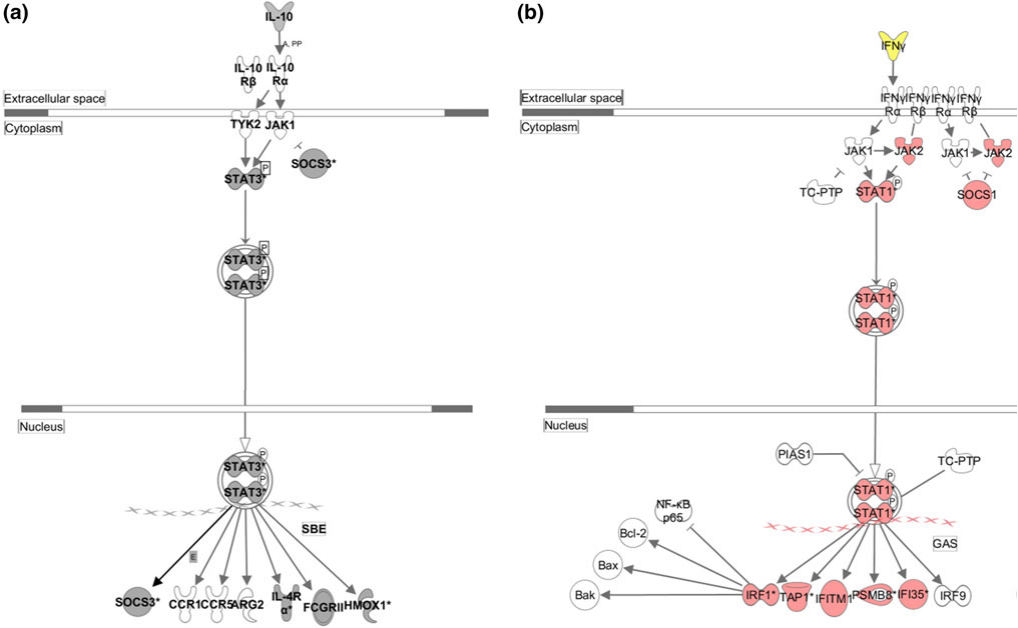
Results of ingenuity pathway analysis in the heart 1668-thioate primed WT mice 3 h after reperfusion. **a** IL-10-dependent suppressive target genes were significantly elevated (*p* < E−4; *gray color*). **b** Significant mRNA up-regulation of constituents involved in the IFNγ pathway (*p* < E−4; *red color*). A minimum of three animals per group were independently analyzed (stringency cut-off: *p* < 0.0001 above controls, up-regulated mRNAs are depicted in *gray* or *red color*)

Ingenuity pathway analysis (IPA) was used to identify the pattern of regulatory interactions underlying 1668-thioate-dependent gene activation after I/R. 1668-thioate priming in conjunction with I/R up-regulated specific functional groups (Table 1). The analysis of functional groups of activated genes revealed that “cellular movement” (*p* < 4.1E−07) and “inflammatory response” (*p* < 4.68E−07) were markedly induced. Regarding up-regulated pro-inflammatory pathways, the “pattern recognition receptor pathway” (*p* < 3.4E−10), “interferon pathway” (*p* < 1.1E−8) and the “IL-6 pathway” (*p* < 2.4E−7) were most prominent (Table 2). Therefore, up-regulated genes of the interferon pathway are displayed in detail in Fig. 7b. Among the elevated decisive genes of the interferon pathway is the transcription factor STAT1, which in turn induces the STAT1-dependent protein IRF1 thereby inhibiting NFκBp65. At the same time, the anti-inflammatory IL-10 pathway (Fig. 7a) was significantly up-regulated in the microarray analysis (*p* < E−8, Table 2). This was in concordance with RT-qPCR analysis that revealed a massive up-regulation in IL-10 mRNA (Fig. 6g).

**Table 1:** Microarray analysis 3 h after reperfusion: results from analysis of the top 5 up-regulated functional groups

Relevant functional groups		
ID	Functional group	*p* value
1	Cellular growth and proliferation	4.7E−07
2	Inflammatory response	4.68E−07
3	Cellular movement	4.1E−07
4	Immune cell trafficking	3.3E−07
5	Hematological system development and function	3.0E−07

**Table 2:** Microarray analysis 3 h after reperfusion: results from analysis of the top 5 up-regulated inflammatory signaling pathways

Relevant inflammatory pathways		
ID	Inflammatory signaling pathway	*p* value
1	Pattern recognition receptor pathway	3.4E−10
2	IL-10 pathway	6.5E−08
3	Interferon signaling	1.1E−08
4	Complement system	3.1E−06
5	IL-6 pathway	2.4E−07

A minimum of 3 animals per group were independently analyzed (stringency cut-off: *p* < 0.0001 above controls)

#### Analysis of cardiac function

**Fig. 8:**
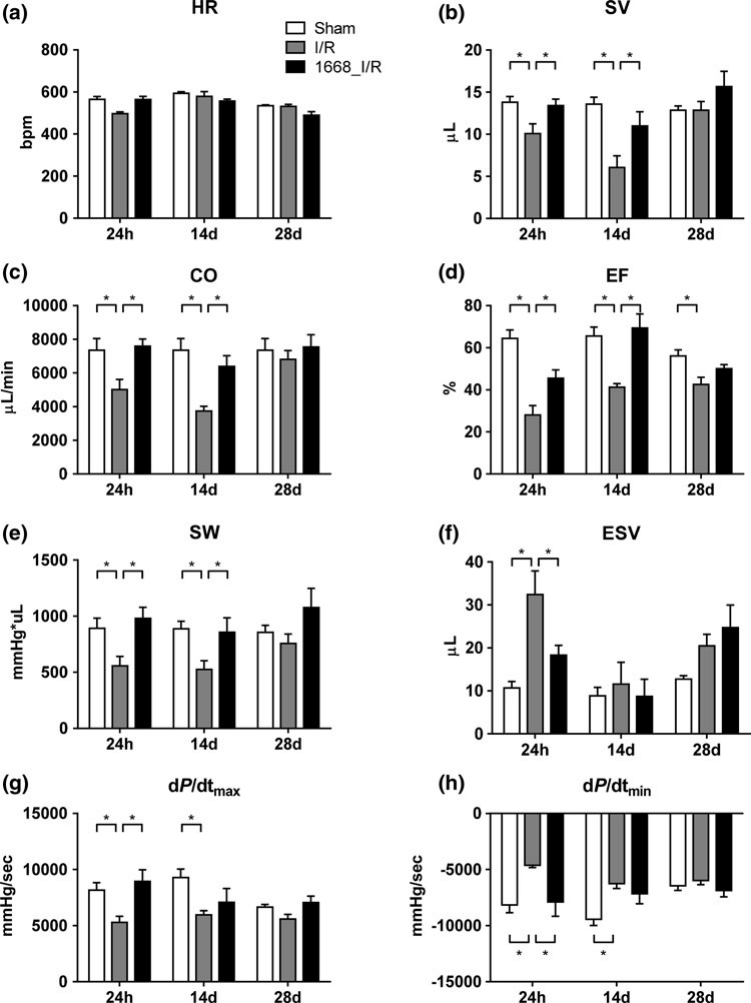
Hemodynamic parameters, **a** heart rate (HR), **b** stroke volume (SV), **c** cardiac output (CO), **d** ejection fraction (EF), **e** stroke work (SW), **f** end-systolic volume (ESV), **g** velocity of pressure increase (d*P*/d*t*_max_), **h** velocity of pressure decrease (d*P*/d*t*_min_) after 24 h, 14 days and 28 days of reperfusion (*n* = 10, *p < 0.05). After 24 h of reperfusion, I/R leads to a significant impairment of all measured parameters except HR, which was prevented by priming with 1668-thioate. After 14 days, the effect of 1668-thioate priming was still maintained

Functional parameters characterizing the cardiovascular system were measured 24 h, 14 days and 28 days after I/R with a pressure–volume catheter (Fig. 8a–h). An obvious pattern of response was that all recorded parameters [heart rate (HR), stroke volume (SV), cardiac output (CO), ejection fraction (EF), stroke work (SW), end-systolic volume (ESV), velocity of pressure increase (d*P*/d*t*_max_) and of pressure decrease (d*P*/d*t*_min_)] were impaired 24 h after I/R in the animals without pre-conditioning. Priming with 1668-thioate prevented at least partially this deterioration of cardiac function. This general pattern was preserved at least until 14 days. 28 days after I/R, the cardiac function of animals without pre-conditioning had largely recovered to the level of the 1668-thioate primed mice.

## Discussion

The objective of this study was to characterize local and systemic inflammatory responses to CpG-ODN pre-conditioning before and after cardiac I/R injury and thus to elucidate how the CpG-ODN 1668-thioate can protect the heart from I/R injury and preserve cardiac function. The most important signaling pathways were visualized via micro-array technique of regulated genes. Finally, the functional consequences of priming with 1668-thioate were monitored by measuring hemodynamic characteristics in vivo.

During pre-conditioning, pro-inflammatory mediators such as TNF-α, IL-1β and IL-6 increased in a time-dependent manner in the blood and in the heart. For the explanation of the beneficial effect of pre-conditioning, the level of pro-inflammatory mediators directly before the onset of ischemia seems to be most relevant. Interestingly, TNF-α was the only pro-inflammatory cytokine, which was still significantly elevated 16 h after priming. IFNγ was extremely high in the blood just before the ischemic insult. However, an early and prominent up-regulation of IFNγ can induce regulatory proteins like interferon regulatory factors (IRF). In this context, Stevens et al. demonstrated that protection from I/R brain injury after priming with CpG-ODNs requires IFR3 and IRF7 [40]. In our hands, IFNγ reached its maximum 16 h after priming, thereby following the preceding up-regulation of IL-12. This observation can be explained as IFNγ is produced in leukocytes like NK cells and T-cells on stimulation with IL-12 and IL-18 [20, 36]. In the blood and the heart, the anti-inflammatory mediator IL-10 was at its highest level 6 and 16 h after priming with 1668-thioate, i.e. at the onset of cardiac ischemia, anti-inflammatory mediators were well above control levels. Neither priming with 1612-thioate nor with H154-thioate induced any significant change in inflammatory mediators during the priming phase underlining the lack of inflammatory potency of these ODNs [23]. Priming-induced increases of cytokines were absent in the blood and hearts of TLR9-D mice proving their dependency on TLR9 signaling. Consequently, infarct size in TLR9-D animals was insensitive to priming with 1668-thioate.

In a study with CpG-ODN 1826 pre-conditioning, Cao et al. [7] interrupted the TLR9-signaling further downstream during priming, i.e. blocking PI3K/Akt and thus, also prevented the pre-conditioning influence of CpG-ODNs. This branch of the TLR9-signaling pathway seems to be common with the TLR2 signaling as the same inhibition strategy also prevented pre-conditioning via TLR2 [15].

Micro-array analysis during the priming phase supported the findings of RT-qPCR. For better comparison with earlier results of Mathur et al. [28], we also focused on NF-κB pathway inhibitors. Re-calculation of these data to fold-change revealed a somewhat higher up-regulation of the gene expression of TNFAIP3, NFKBIA, TRIM30 in our hands (Mathur vs. own data: TNFAIP3: 2.6 vs. 3.0; NFkBIA: 2.2 vs. 3.7; TRIM30: 3.8 vs. 5.04). Concurrently those investigations found that TNIP1 was not elevated. Hence, switching on NF-κB pathway inhibitors during pre-conditioning with CpG-ODNs seems to be a relatively stable phenomenon, which contributes to cardiac protection.

However, in our hands the most obvious effect of pre-conditioning was the up-regulation of IL-10 as demonstrated in the RT-qPCR. The micro-array analysis performed after pre-conditioning with 1668-thioate also detected a significant albeit less prominent up-regulation compared with the interferon pathway (IL-10 pathway: 2.1E−3 vs. IFN signaling pathway: 1.2E−11). However, the micro-array analysis may underscore the role of the IL-10 pathway during priming, as this analysis was performed 4 h after application of 1668-thioate, i.e. at a time point when IL-10 was still in the rising phase according to RT-qPCR.

16 h after priming with 1668-thioate, GR-1^high^/(F4/80)^high^ monocytes were significantly elevated in the blood, whereas GR-1^low^/(F4/80)^high^ reparative monocytes [13] had not been influenced by pre-conditioning. Possibly, the elevated inflammatory GR-1^high^/(F4/80)^high^ monocytes were able to invade the infarct area faster than without pre-conditioning. In addition, a switch from GR-1^high^/(F4/80)^high^ to reparative GR-1^low^/(F4/80)^high^ may be promoted by the raised IL-10 concentration in the blood and in the heart [2].

24 h after I/R, significant differences in troponin T levels in the blood between control (d-GalN alone) and 1668-thioate primed animals were detected (Fig. 5a, b). This can be taken as an indication of differences in infarct size. Possibly, this difference in troponin T levels is present much earlier as Mathur et al. [28] found differences in troponin I already 6 h after reperfusion in a mouse model. The early appearance of differences in troponin prompted us to monitor the levels of inflammatory mediators in blood and heart 30 min and 3 h after I/R. Interestingly, I/R by itself did not enhance any systemic marker of inflammation in the blood at the investigated time points. However, animals primed with 1668-thioate exhibited increased levels of TNF-α, IL-1β, IL-10, IL-12, and IFNγ either at both or only at one time point after reperfusion (Fig. 5c–h). Pro-inflammatory cytokines were increased mainly at the early time point, whereas IL-10 seemed to become up-regulated delayed.

In the heart, mRNA expression of TNF-α, IL-1β, IL-6, and IL-10 did only partially reflect the situation in the blood. Again none of the cytokines was induced by I/R alone. TNF-α, IL-6 and IL-10 were increasingly expressed over time, whereas IL-1β did not reach the level of significance at any time point. Interestingly, 1668-thioate priming had raised IL-10 mRNA expression by a factor of 200 until the start of I/R. 30 min after reperfusion, IL-10 mRNA expression was already more than doubled compared to the start of I/R. At 3 h after reperfusion, the IL-10 mRNA had again increased two times, i.e. IL-10 was 1,000× higher than in untreated hearts at this time. Taken together, there were signs of an increased inflammation after I/R in the blood and the heart of mice pre-treated with 1668-thioate. IL-6, which has been reported to exhibit pro- as well as anti-inflammatory properties [34], was expressed relatively high in this group. However, the expression of the anti-inflammatory cytokine IL-10 in the heart after priming with 1668-thioate and I/R outbalanced all other investigated mediators by far. It should be noted that the source of the pro- as well as anti-inflammatory cytokines was not directly investigated here. Thus, cytokines may derive from either immune cells or sedentary cells of the heart.

Micro-array analysis performed in this study (Fig. 7, Tables 1, 2) revealed that I/R itself increased expression in a relatively small number of genes further supporting the results from the RT-qPCR analysis. In contrast, 1668-thioate pre-treatment in conjunction with cardiac I/R resulted in significant up-regulation of 635 genes 30 min after reperfusion and persisted towards 3 h. The massive, CpG-ODN priming-dependent up-regulation of gene expression after I/R can be taken as a sign of increased inflammation. However, the extremely high expression of IL-10 mRNA demonstrated by RT-qPCR is further supported by the pathway analysis as different downstream components of IL-10 signaling had been up-regulated consequently (Fig. 7a). Furthermore, some of the initially pro-inflammatory pathways finally lead to the induction of suppressive networks, which terminate the inflammatory processes, e.g. IFNγ up-regulation induces IRF as mentioned above. Interestingly, IL-10 has recently been shown to be the central mediator of remote ischemic pre-conditioning of the heart [5]. As remote pre-conditioning up-regulated IL-10 in the ischemic organ, this IL-10 was able to protect the heart against I/R injury. This finding was further confirmed by the fact that IL-10 deficient mice could not profit from remote ischemic pre-conditioning. In the present study, we found that priming with CpG-ODN massively up-regulated IL-10 in the blood as well as in the heart. This was seen during the priming phase and even more extensively after I/R. As Cai et al. demonstrated that application of IL-10 prior to I/R induced phosphorylation of Akt, the massive increase of IL-10 observed in our hands may be the clue to the Akt-phosphorylation, which was shown by Cao et al. [7] to be a reason for smaller infarct size after CpG pre-conditioning. Interestingly, IL-10 up-regulation seems also to attenuate cardiac remodeling after I/R injury, which may improve cardiac long-term performance [33].

Pre-treatment with 1668-thioate in our study led to a significant reduction of IS of more than 75 % (Fig. 6a). The amount of IS reduction seems to be pronounced, but Ha et al. also obtained a distinct reduction of infarct size (65 %) due to LPS priming using a comparable I/R protocol [16]. Recently, Cao et al. [7] reported a smaller reduction of infarct size by about 35 % in an open-chest model after CpG-ODN priming. The higher degree of cardiac protection obtained in our hands may be attributed to differences in the applied CpG-ODNs (1668-thioate vs. 1826), the duration of priming and/or reperfusion (16 h/24 h vs. 1 h/4 h) as well as to our superior surgery model (closed-chest vs. open-chest model) [22, 26]. In a further study on myocardial I/R, where the effect of pre-conditioning with CpG-ODN 2395 was estimated by troponin I measurement [28], pre-conditioning induced lower troponin I levels in the blood indicating reduction of infarct size. Taken together, three study groups reported a cardio-protective influence of pre-conditioning with CpG-ODNs using different ODNs and diverse time-frames. Thus, the ODN-dependent cardio-protection seems to be a stable mechanism. Here, we show that this effect was TLR9-dependent as 1668-thioate priming did not affect infarct volume in TLR9-D animals. However, the TLR9-deficiency by itself reduced infarct size by about 35 % (Fig. 6a). This phenomenon reported here for the first time needs further studies to be explained. To confirm the above-mentioned hypothesis that IL-10 up-regulation is an essential step for the beneficial effect of ODN-priming we interrupted the IL-10-dependent signaling pathway by block of IL-10R1 with an appropriate antibody. This intervention caused a significant increase of infarct size, which may be attributed to block of physiological amounts of IL-10. In the case of IL-10R1 inhibition, the effect of previous ODN-priming on infarct size was completely prevented. Taken together, IL-10 seems to be the essential regulator, which transmits cardioprotection following ODN-priming. All cells in the heart, i.e. immune cells as well as sedentary cells like cardiomyocytes and cardiofibroblasts, may be influenced by IL-10. However, the cellular target of IL-10 was not elucidated in the present study but shall be pursued in further investigations.

After myocardial I/R, a decline in cardiac functional parameters is expected [48]. Mice suffering from I/R exhibited smaller SV, CO, EF, SW, d*P*/d*t*_max_ and d*P*/d*t*_min_ at 24 h and 14 days of reperfusion as well as an increased ESV at 24 h. This shows that the ischemic event elicited in our experiments is sufficient to cause the anticipated pathology. Interestingly, all mentioned parameters were superior to the I/R group if the animals had been primed with 1668-thioate. This beneficial influence of CpG-ODN pre-conditioning lasted until 14 days after I/R. Most of the measured characteristics remained nearly identical to sham animals in 1668-thioate pre-treated mice although they had suffered from 1 h of ischemia. Interestingly, other groups using different ODNs and different times for pre-conditioning found comparable improvements of cardiac function after I/R by application of ultrasound techniques [7, 28]. Only the present investigation demonstrates that during remodeling, the resting performance of the hearts in the I/R group recovered reaching the level of the 1668-thioate I/R group 28 days after reperfusion.

Taken together, three different CpG-ODNs (1668-thioate, 1826 [7] and 2395 [28]) applied in a different time-frame for priming (16 h, 1 h [7], 24 h [28]) and in different studies all led to successful pre-conditioning. Thus, it may be speculated that there is a common mechanism, by which CpG-ODNs induce effective organ protection. As priming with CpG-ODNs rapidly leads to a long-lasting systemic inflammation, the time-window for pharmacological pre-conditioning seems to be wide. The increase of IL-10 reported here for the first time may be regarded as the common mechanism for pre-conditioning in all three studies. In an investigation with ischemic pre-conditioning, IL-10 administration has been shown to protect the heart against I/R injury via PI3K/Akt signaling [5]. CpG-ODN-dependent cardioprotection could also be prevented by disruption or block of the PI3K/Akt signaling pathway [7]. Thus, the remaining link to be elucidated was whether the CpG-ODN-dependent activation of PI3K/Akt signaling is dependent on IL-10 up-regulation. The present study shows for the first time that priming with a synthetic CpG-ODN massively increases IL-10 expression in the blood and in the heart before and after I/R. In a reverse approach, IL-10 signaling was inhibited and consequently, infarct size rose and ODN-priming remained ineffective. Therefore, the mechanism, by which CpG-ODN is able to protect the heart, seems to be the induction of IL-10 expression and a consecutive activation of the PI3K/Akt pathway.

A transfer of these new findings from mice to humans may be hampered by differences in inflammatory responses between both species [37]. However, a correlation between the anti-inflammatory cytokine IL-10 and acute myocardial infarction in humans has been shown [9]. Furthermore, pre-conditioning as well as exercise seems to induce cardioprotective factors in humans, which are able to overcome the inter-species differences as they were able to decrease infarct size and cardiac function in isolated rat hearts [31]. Although pre-conditioning is not suited to reduce I/R injury in case of a sudden myocardial infarction, it may be applied prior to elective cardiac surgery. Here, application of remote ischemic pre-conditioning (RIPC) to humans before cardiac surgery has induced indications of cardioprotective effects [1]. Furthermore, RIPC is actually being evaluated in a multi-center clinical trial [30]. The cardioprotective influence of IL-10 demonstrated in this and other studies may also be transferred to the clinical setting by direct application of IL-10 prior to cardiac surgery.

## Electronic supplementary material

Below is the link to the electronic supplementary material.

Supplementary material (DOCX 18 kb)

**Supplement Figure 1** Cytokine (a-e: TNF-α, IL-1β, IL-6, IL-12) and interferon-γ (f) proteins in blood serum of WT and TLR9-D mice measured at 2, 4, 6 and 16 h after priming with 1668-thioate. All investigated mediators were up-regulated during the priming phase by 1668-thioate stimulation in WT but not in TLR9-D mice (n = 5/group, *: p < 0.05). **Supplement Figure 2.** RT-qPCR of mRNA expression of PRRs (a, b) and cytokines (c-f) in the heart of WT and TLR9-D mice measured at 2, 4, 6 and 16 h after priming with 1668-thioate. Stimulation with 1668-thioate differentially up-regulated TLR2 and -9 and the cytokines TNF-α, IL-1β, IL-6, and IL-10 during the priming phase in WT but not in TLR9-D mice (n = 8/group, *: p < 0.05) (DOCX 83 kb)
