# Supplementary material for: Pre-conditioning with synthetic CpG-oligonucleotides attenuates myocardial ischemia/reperfusion injury via IL-10 up-regulation
Source: Basic Res Cardiol. 2013 Aug 9;108(5):376. doi: 10.1007/s00395-013-0376-7 (PMC3778842; doi:10.1007/s00395-013-0376-7)
Supplement: Supplementary file 1 — Supplementary material (DOCX 18 kb) [file 395_2013_376_MOESM1_ESM.docx]

**Supplement Material and Methods**

**Ischemia/Reperfusion-Surgery**

The ligation of the LAD was performed under general anesthesia as a *closed chest* model, to exclude inflammatory induction due to the surgical procedure [[22](#_ENREF_22), [35](#_ENREF_35)]. Mice were anesthetized with isoflurane 2.5 Vol% via a nasal cone (Forene, Abbott GmbH, Wiesbaden, Germany). Anesthesia depth was confirmed by squeezing paws, body temperature and electric cardiac activity (ECG) were monitored. The nose was placed in a nasal cone which was connected to an isoflurane vapor to maintain anesthesia during the intubation procedure. The head was slightly reclined by fixing the upper incisors with a 6-0 suture to facilitate intubation. Ventilation was confirmed by observing thorax excursions and adopted to physiological parameters (Minivent, HUGO SACHS ELEKTRONIK-HARVARD APPARATUS GmbH, March-Hugstetten, Germany, respiratory rate 105*min-1, 200 μl tidal volume). A left parasternal incision was performed between the third and the fourth rib and an 8-0 prolene suture was passed underneath the LAD. Both ends of the suture were threaded through a 1 mm section of a poly-ethylene (PE) tube and both ends of the suture were tightened to confirm the correct position by emerging paleness of the distal myocardium. Afterwards, both ends of the suture were released again, formed into a knot, placed subcutaneously, and the chest was closed with 6-0 sutures. Anesthesia was turned off, mice were extubated and regained consciousness while breathing 100% O_2_. Analgesia was obtained by s.c. application of buprenorphine (0.1 mg/kg s.c.). After a recovery period of 5-7 d I/R experiments were performed. Mice were sedated with propofol (1% in 0.2 ml; 2 mg i.p.) allowing spontaneous breathing in the supine position. If necessary the propofol injection was repeated. Again body temperature and ECG (PowerLab, ADInstruments GmbH, Spechbach, Germany) were recorded. The former skin incision was re-opened and the subcutaneously located thread could now be manipulated without opening the chest. Then tension was carefully applied to the loop to achieve an ischemia visible as a significant ST-elevation. 1 h after ischemia, the loop was released and reperfusion induced the recession of the ST-segment elevation. Finally, the skin was closed.

Various experimental procedures were performed at different time-spans of reperfusion (Figure 1).

**Assessment of Infarct Size**

The determination of the IS was performed 24 h after I/R. After the ligation of the LAD, 10% phtalo blue (Heubach, Germany) was injected into the left atrium in order to determine the area at risk (AAR). The hearts were excised, cut into 1 mm-thick transverse slices and incubated with 1.5% triphenyltetrazolium chloride (TTC, Sigma-Aldrich Chemie GmbH, Munich, Germany) in order to visualize the infarct area (IA). Phtalo blue stained non area at risk (NAAR), AAR and IS were quantified with ImageJ (Version 1.29, NIH, USA). This protocol has been described elsewhere.
