# Supplementary material for: Pre-conditioning with synthetic CpG-oligonucleotides attenuates myocardial ischemia/reperfusion injury via IL-10 up-regulation
Source: Basic Res Cardiol. 2013 Aug 9;108(5):376. doi: 10.1007/s00395-013-0376-7 (PMC3778842; doi:10.1007/s00395-013-0376-7)

Supplement Figure 1

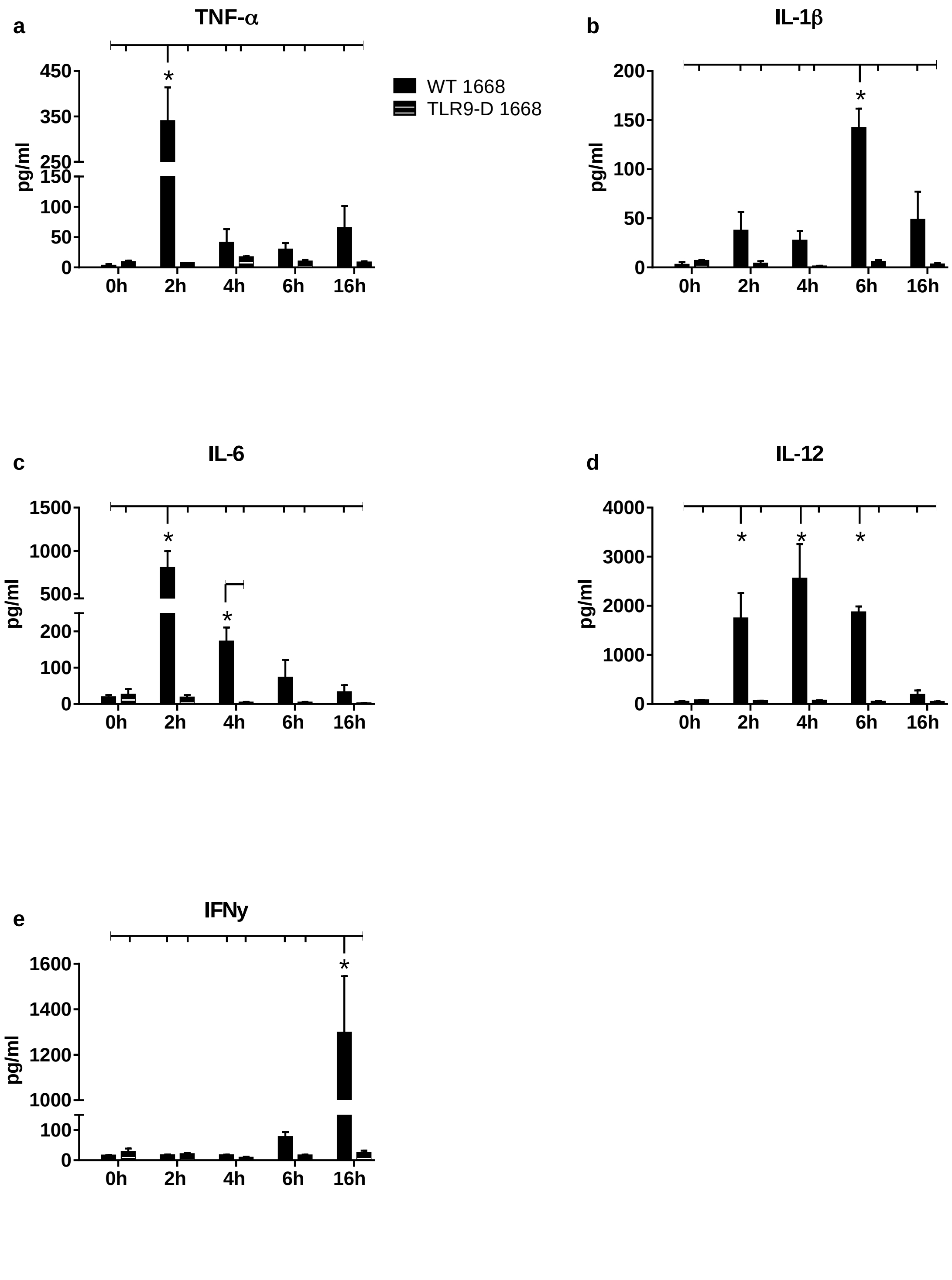

Supplement Figure 2

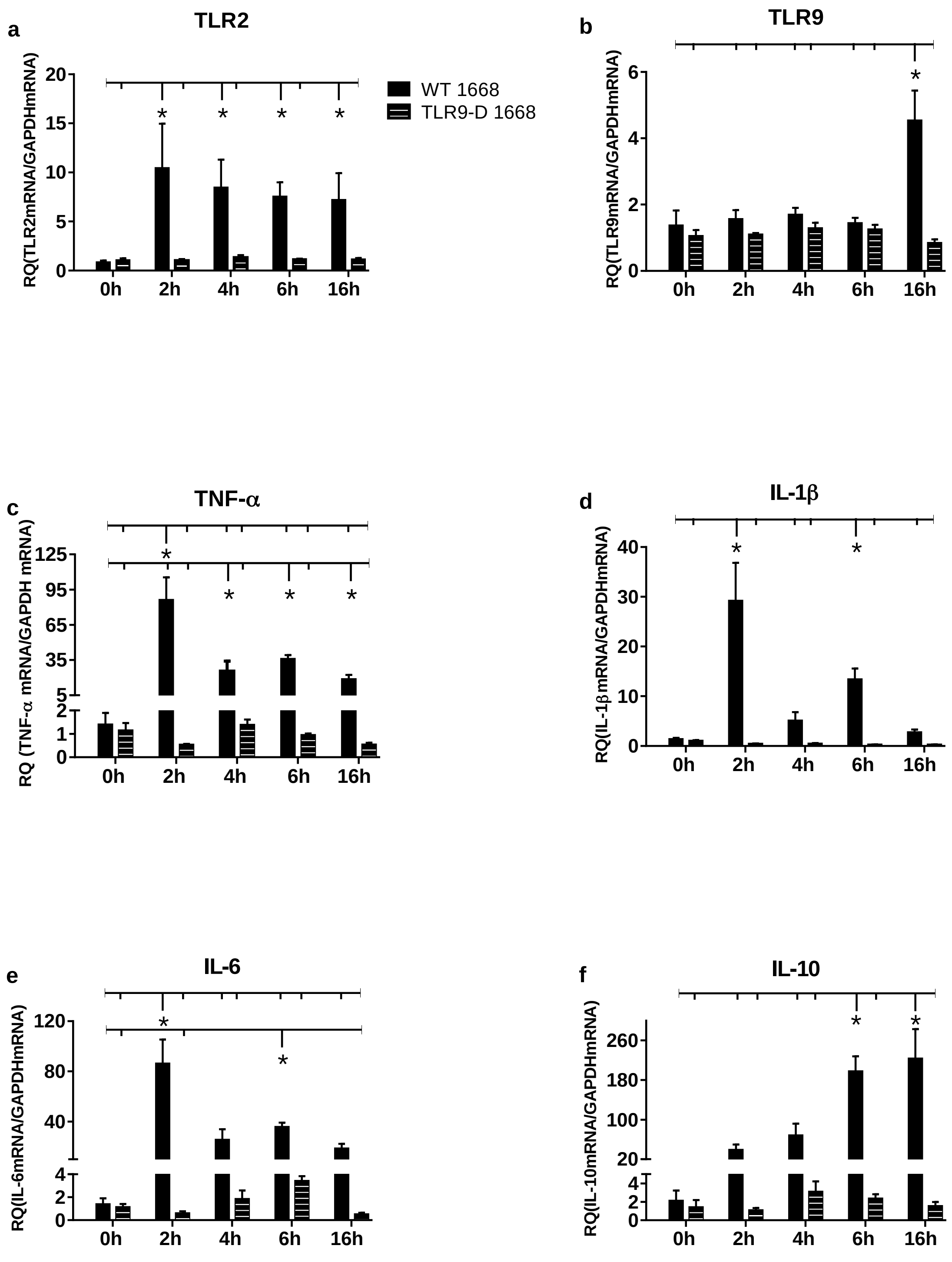

Supplement: Supplementary file 2 [file 395_2013_376_MOESM2_ESM.docx]
